# VISTA Trials

**DOI:** 10.1186/2043-9113-5-S1-S4

**Published:** 2015-05-22

**Authors:** Caroline Gilotay, Stéphane Lejeune

**Affiliations:** 1 EORTC, Avenue Emmanuel Mounier 83/11, 1200 Brussels, Belgium

## Characterisation

Tool, Clinical Data Management System, randomization.

## Description

VISTA Trials is a professional, affordable and efficient Clinical Data Management System (CDMS) developed by the European Organisation for Research and Treatment of Cancer (EORTC). It will be available to research organizations looking for a solution to run their multinational clinical trials. VISTA Trials has been used by EORTC for many years and is now being upgraded to CDISC standards and ECRIN requirements. It is a fully integrated and web-based solution applicable to all therapeutic areas. VISTA Trials is composed of 4 modules built around different functionalities: TrialDesign for database and intelligent eCRF design (with built-in logic), Clinical Data for patient inclusion and randomization, electronic data capture, monitoring and data validation, Rights and Roles for access rights and user management, and Reports for data exports and metrics. Version management will be part of the system, making protocol amendments easy to handle. The system is based on CDISC standards, employing ODM as the backbone for the VISTA data model. Thanks to the ODM import and export elements, it is possible to handle any trial defined in that format, including libraries that will be a component of VISTA trials facilitating the management of user’s standard CRFs. Dictionaries such as MedDRA and CTCAE as well as blinding and unblinding capacities will be integrated in the software in its second version.

VISTA Trials is GCP and 21CFR part 11 compliant, validated by a risk based approach following GAMP 5 guidance. Version 1.0 of the software is planned to be released by June 2015 with a SaaS deployment model.

## Status of development

In development, release expected by June 2015.

## Users

Academic research organisations.

## Links

http://www.vistatrials.org/

**Figure 1:**
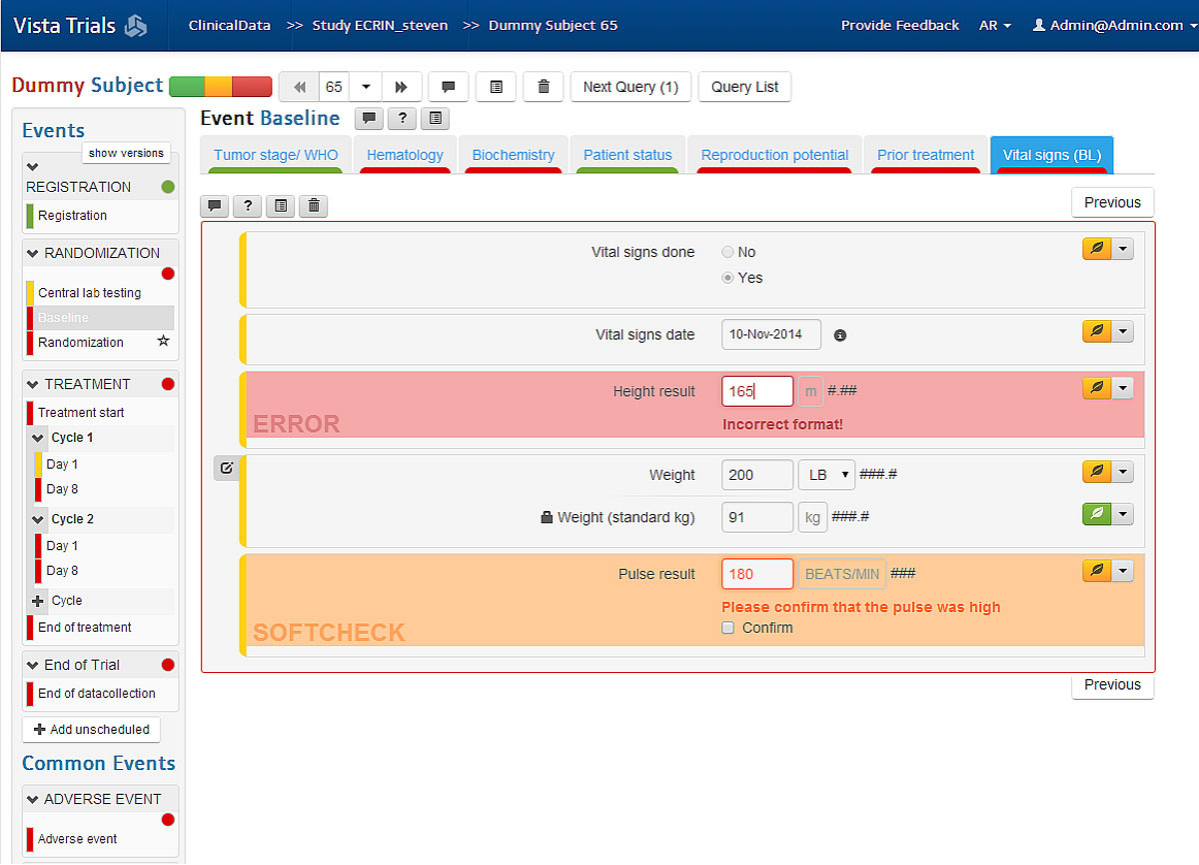
VISTA Trials user interface. Display of ClinicalData module indicating a check for correctness of data input (in red).

